# Glycogen production for biofuels by the euryhaline cyanobacteria *Synechococcus* sp. strain PCC 7002 from an oceanic environment

**DOI:** 10.1186/1754-6834-7-88

**Published:** 2014-06-11

**Authors:** Shimpei Aikawa, Atsumi Nishida, Shih-Hsin Ho, Jo-Shu Chang, Tomohisa Hasunuma, Akihiko Kondo

**Affiliations:** 1 Department of Chemical Science and Engineering, Graduate School of Engineering, Kobe University, 1-1 Rokkodai, Nada, Kobe 657-8501, Japan; 2 Core Research for Evolutional Science and Technology, Japan Science and Technology Agency, 3-5 Sanban, Chiyoda, Tokyo 102-0075, Japan; 3 Organization of Advanced Science and Technology, Kobe University, 1-1 Rokkodai, Nada, Kobe 657-8501, Japan; 4 Department of Chemical Engineering, National Cheng Kung University, No.1 University Road, Tainan 701, Taiwan; 5 Research Center for Energy Technology and Strategy, National Cheng Kung University, No.1 University Road, Tainan 701, Taiwan; 6 Center for Bioscience and Biotechnology, National Cheng Kung University, No.1 University Road, Tainan 701, Taiwan; 7 Precursory Research for Embryonic Science and Technology (PRESTO), Japan Science and Technology Agency, 3-5 Sanban, Chiyoda, Tokyo 102-0075, Japan; 8 Biomass Engineering Program, RIKEN, 1-7-22 Suehiro, Tsurumi, Yokohama 230-0045, Japan; 9 Department of Food Bioscience and Technology, College of Life Sciences and Biotechnology, Korea University, Seoul 136-713, Republic of Korea

**Keywords:** Carbon source, Cyanobacteria, Glycogen, Salinity, S*ynechococcus* sp. strain PCC 7002

## Abstract

**Background:**

Oxygenic photosynthetic microorganisms such as cyanobacteria and microalgae have attracted attention as an alternative carbon source for the next generation of biofuels. Glycogen abundantly accumulated in cyanobacteria is a promising feedstock which can be converted to ethanol through saccharification and fermentation processes. In addition, the utilization of marine cyanobacteria as a glycogen producer can eliminate the need for a freshwater supply. *Synechococcus* sp. strain PCC 7002 is a fast-growing marine coastal euryhaline cyanobacteria, however, the glycogen yield has not yet been determined. In the present study, the effects of light intensity, CO_2_ concentration, and salinity on the cell growth and glycogen content were investigated in order to maximize glycogen production in *Synechococcus* sp. strain PCC 7002.

**Results:**

The optimal culture conditions for glycogen production in *Synechococcus* sp. strain PCC 7002 were investigated. The maximum glycogen production of 3.5 g L^−1^ for 7 days (a glycogen productivity of 0.5 g L^−1^ d^−1^) was obtained under a high light intensity, a high CO_2_ level, and a nitrogen-depleted condition in brackish water. The glycogen production performance in *Synechococcus* sp. strain PCC 7002 was the best ever reported in the α-polyglucan (glycogen or starch) production of cyanobacteria and microalgae. In addition, the robustness of glycogen production in *Synechococcus* sp. strain PCC 7002 to salinity was evaluated in seawater and freshwater. The peak of glycogen production of *Synechococcus* sp. strain PCC 7002 in seawater and freshwater were 3.0 and 1.8 g L^−1^ in 7 days, respectively. Glycogen production in *Synechococcus* sp. strain PCC 7002 maintained the same level in seawater and half of the level in freshwater compared with the optimal result obtained in brackish water.

**Conclusions:**

We conclude that *Synechococcus* sp. strain PCC 7002 has high glycogen production activity and glycogen can be provided from coastal water accompanied by a fluctuation of salinity. This work supports *Synechococcus* sp. strain PCC 7002 as a promising carbohydrate source for biofuel production.

## Background

Currently, biorefinery, including production of biofuels and bio-based chemicals, has received considerable attention. Additionally, environmental concerns and the depletion of oil reserves have resulted in promoting research on more environmentally benign and sustainable biofuels such as bioethanol.

Oxygenic photosynthetic microorganisms, including cyanobacteria and microalgae, have attracted attention as an alternative carbon source for biorefineries [1-3]. Cyanobacteria and microalgae convert solar energy to biomass more efficiently (0.5 to 2.0% efficiency) than energy crops such as switchgrass (0.2% efficiency) [4], and their α-polyglucans such as glycogen from cyanobacteria or starch from microalgae, can be converted to bioethanol by yeast fermentation [5-9]. In addition, they are capable of growing in aquatic environments, providing the additional benefit of whole-year cultivation using non-arable land. In particular, the cultivation of cyanobacteria and microalgae using seawater or brackish water eliminates the impact on freshwater resources [10]. These carbohydrate-producing species need to tolerate a wide salinity range because the salinity of coastal water fluctuates with changes in freshwater inflow by climate, weather, and diurnal tidal current. Therefore, in the current study, the euryhaline cyanobacteria *Synechococcus* sp. strain PCC 7002, which is well-suited for growing in a coastal region, was selected as a carbohydrate producer. *Synechococcus* sp. strain PCC 7002 is naturally transformable and its genome has been fully sequenced [11]. Based on these superior characteristics, *Synechococcus* sp. strain PCC 7002 is a model organism for research on cyanobacterial metabolites and is expected to be a platform for biotechnological applications by metabolic engineering [12-17].

According to definition, glycogen productivity is estimated from glycogen content and biomass productivity. To improve glycogen productivity in cyanobacteria, both the glycogen content and biomass productivity need to be enhanced. In general, glycogen is accumulated via nitrogen depletion in many cyanobacteria species, such as *Synechococcus* sp. strain PCC 7002, *Synechocystis* sp. strain PCC 6803, *Arthrospira platensis*, *Arthrospira maxima*, *Anabaena variabilis*, and *Anacystis nidulans*[16-23]. Unfortunately, high glycogen content is generated under nitrogen depletion which is associated with low biomass productivity [19,23]. Hence, it is important to obtain a high biomass productivity with a satisfactory glycogen content. However, the integral effect of growth conditions on glycogen production in *Synechococcus* sp. strain PCC 7002 has not been fully investigated.

In the present study, the glycogen production activity of euryhaline cyanobacteria *Synechococcus* sp. strain PCC 7002 was examined under several combined growth conditions, including CO_2_ concentration, light intensity, salinity, and nitrate supply.

## Results

### Effect of light intensity and CO_2_ concentration on cell growth

**Figure 1:**
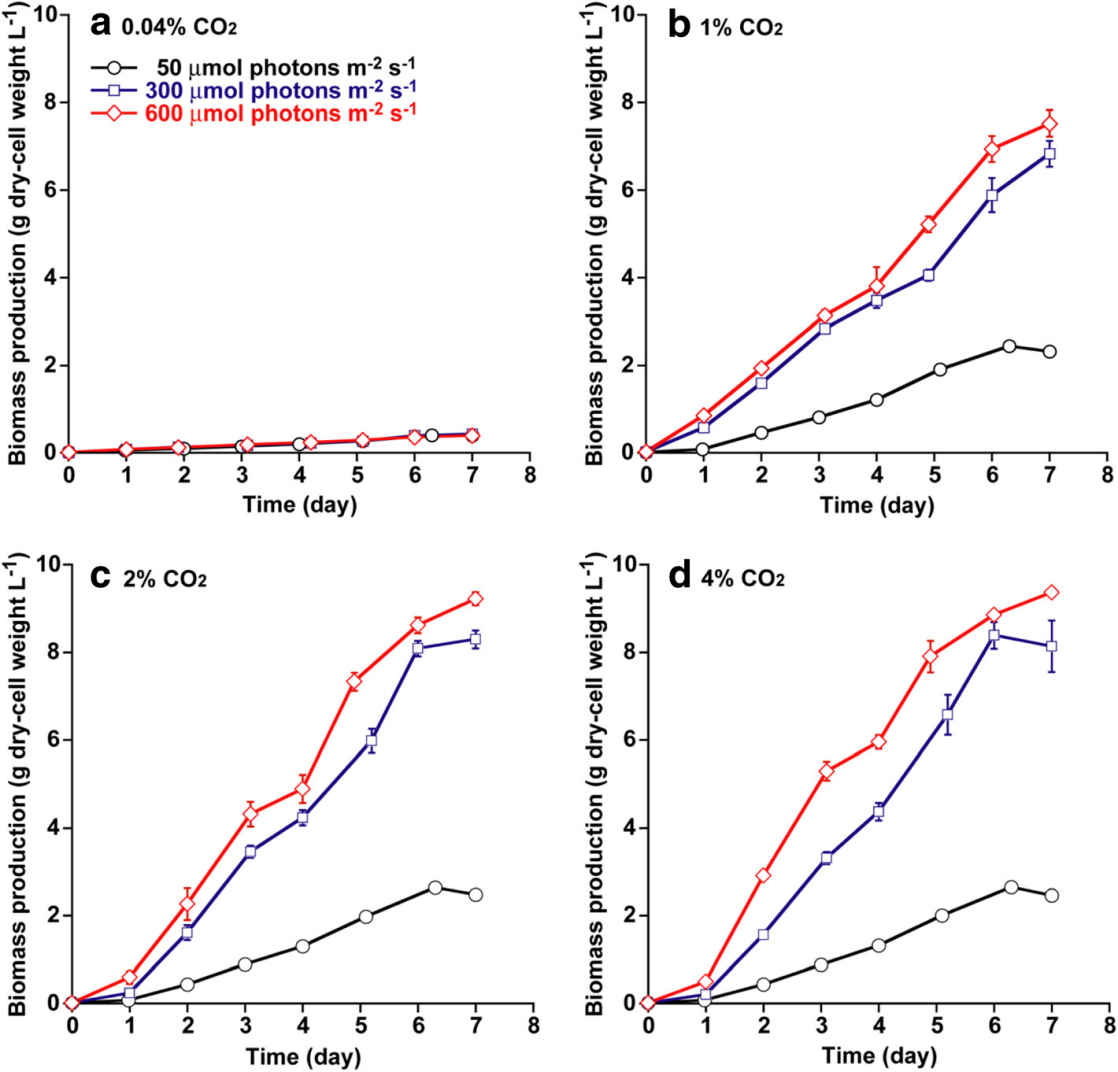
**Growth curve under different light intensities and CO**_**2 **_**concentrations. (a)** Growth curve under 0.04% CO_2_; **(b)**, 1% CO_2_; **(c)**, 2% CO_2_; and **(d)**, 4% CO_2_. Light intensities are 50 (circles), 300 (squares), and 600 μmol photons m^−2^ s^−1^ (diamonds). Error bars indicate standard deviations (SD) of three replicated experiments. In some data points, error bars obtained by three replications are smaller than symbols.

Light intensity and CO_2_ concentration are the key environmental factors for cyanobacterial cell growth [1]. In this study, *Synechococcus* sp. strain PCC 7002 was cultivated on medium A for 7 days under a light intensity of 50 to 600 μmol photons m^−2^ s^−1^ with various CO_2_ concentrations as depicted in Figure 1 (for example, 0.04 to 4% CO_2_ in air). As shown in Figure 1a, cell growth in 0.04% CO_2_ in air (the atmospheric CO_2_ level) was not altered by an increase in light intensity. On the other hand, the cell density of *Synechococcus* sp. strain PCC 7002 tended to increase when increasing CO_2_ concentration from 0.04 to 2% and increasing light intensity from 50 to 600 μmol photons m^−2^ s^−1^. However, further increases in CO_2_ concentration to 4% resulted in no significant difference in cell growth under low and high light intensity, suggesting that excess CO_2_ supply (4%) would not provide a positive effect on cell growth. According to Figure 1, *Synechococcus* sp. strain PCC 7002 cultivated under conditions of high CO_2_ concentration (2 and 4% CO_2_) with high illumination (600 μmol photons m^−2^ s^−1^) reached the highest cell density of around 9 g L^−1^ after 7 days of cultivation. Thus, both enriched CO_2_ supply and high light intensity enhanced the cell growth of *Synechococcus* sp. strain PCC 7002.

### Effect of light intensity and CO_2_ concentration on glycogen content and glycogen production

**Figure 2:**
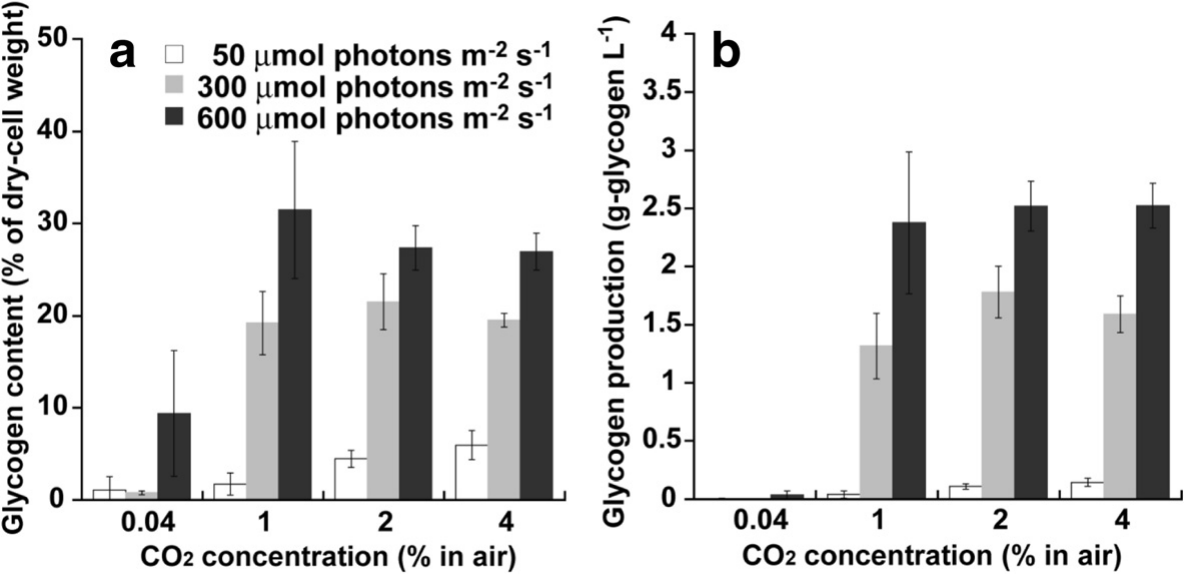
**Glycogen content and glycogen production after 1 week under different light intensities and CO**_**2 **_**concentrations. (a)** Glycogen content; **(b)** glycogen production. Light intensities are 50 (white bars), 300 (gray bars), and 600 μmol photons m^−2^ s^−1^ (Black bars). Data points are mean values from three separate cultures with SD of triplicates.

Light intensity and CO_2_ supply do not only influence the growth of photosynthetic organism but also alter their carbohydrate content [24-26]. Therefore, in this study, the effect of light intensity (50 to 600 μmol photons m^−2^ s^−1^) and CO_2_ concentration (such as 0.04 to 4% CO_2_) on glycogen content were explored, as shown in Figure 2a. Glycogen content increased with an increase in light intensity from 50 to 600 μmol photons m^−2^ s^−1^.

As shown in Figure 2a, the glycogen content under 300 μmol photons m^−2^ s^−1^ increased from 0.8 to 19% as the CO_2_ concentration increased from 0.04 to 1%, and under the same range of CO_2_ concentrations at 600 μmol photons m^−2^ s^−1^, it increased from 9.4 to 31%. However, further increase in CO_2_ concentration to 2% under 300 or 600 μmol photons m^−2^ s^−1^ did not enhance glycogen content.

Glycogen production under 50 to 600 μmol photons m^−2^ s^−1^ in 0.04 to 4% CO_2_ after 7 days was calculated from biomass production and glycogen content, as shown in Figure 2b. The maximum glycogen production of 2.5 g L^−1^ was obtained under 600 μmol photons m^−2^ s^−1^ in 2% CO_2_. Hence, glycogen production in *Synechococcus* sp. strain PCC 7002 was significantly improved by the combined optimization of CO_2_ concentration and light intensity.

### Effect of nitrate supply in different salinity media on glycogen production under high light and high CO_2_ conditions

**Figure 3:**
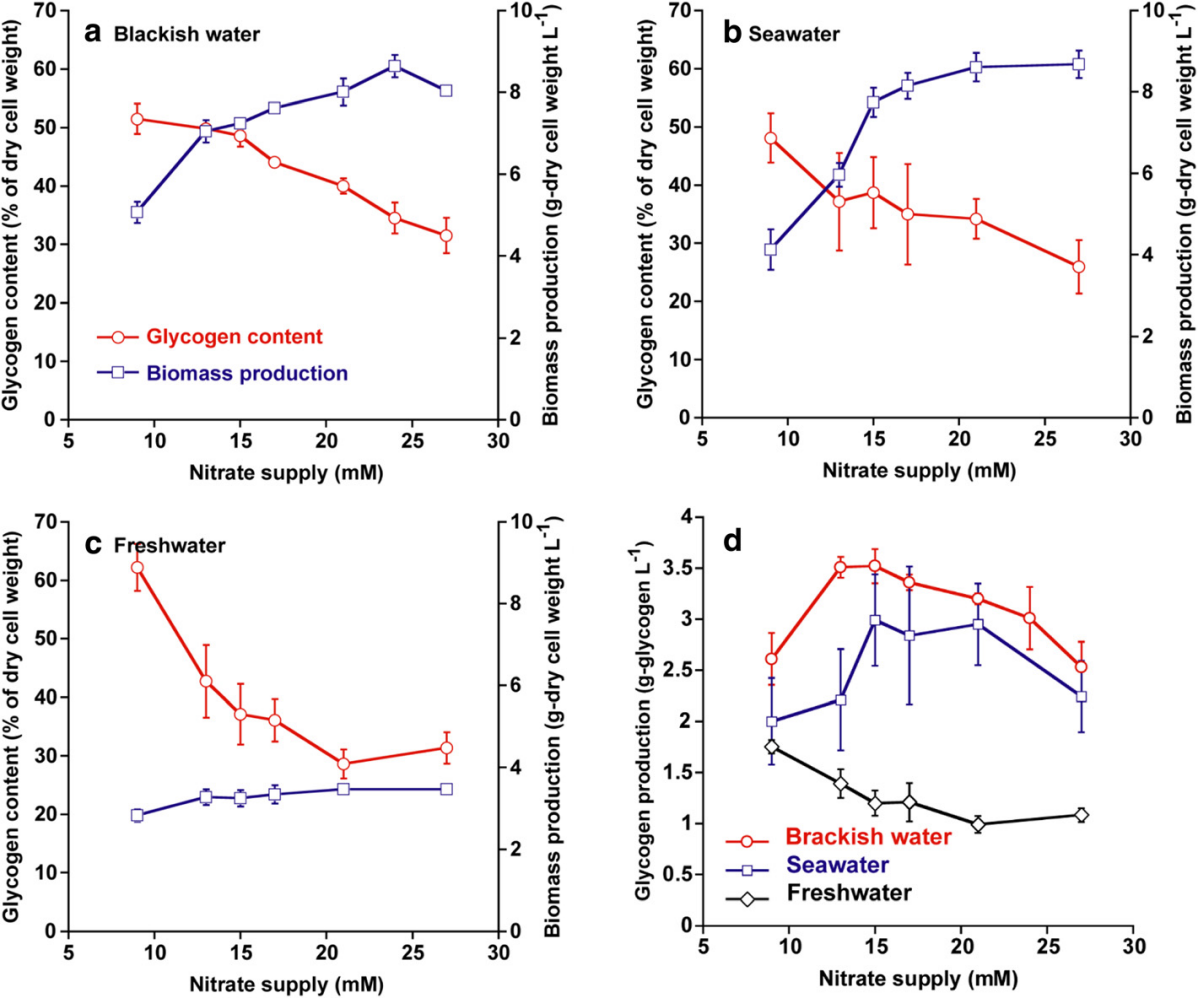
**Biomass production, glycogen content, and glycogen production after 1 week under different salinity conditions. (a)** Biomass production (circles) and glycogen content (squares) in brackish water; **(b)** in seawater; and **(c)** in freshwater; **(d)** glycogen production under different nitrate supplies in brackish water (circles), seawater (squares), and freshwater (diamonds). Cells were cultivated under 600 μmol photons m^−2^ s^−1^ and 2% CO_2_. Data points are mean values from three separate cultures with SD of triplicates.

The accumulation of glycogen occurs in many cyanobacteria, such as *Synechococcus* sp. strain PCC 7002, *Synechocystis* sp. strain PCC 6803, *A. platensis*, *A. maxima*, *A. variabilis*, and *A. nidulans*, under nitrogen-depleted conditions [16-23]. However, high levels of glycogen are generated under nitrogen depletion, which is associated with low biomass productivity [19,23]. Therefore, in this study, the effect of nitrate supply on both glycogen content and biomass production in *Synechococcus* sp. strain PCC 7002 under 600 μmol photons m^−2^ s^−1^ and 2% CO_2_ was investigated. Additionally, in case of cultivation in brackish water or seawater at a coastal region, the salinity of medium was fluctuated according to climate, weather, and diurnal tidal current. Therefore, to estimate the glycogen productivity of *Synechococcus* sp. strain PCC 7002 under different salinity conditions, the glycogen content and biomass production in brackish water (Figure 3a), seawater (Figure 3b), and freshwater (Figure 3c) media were examined. The glycogen content of *Synechococcus* sp. strain PCC 7002 in all media increased with a drop of nitrate concentration from 27 to 9 mM, reaching 52, 50, or 62% of dry-cell weight in brackish water, seawater, or freshwater medium, respectively. Unfortunately, the biomass productions were suppressed below 21 mM in brackish water and below 15 mM in seawater (Figure 3a,b). Thus, in this study, the glycogen production of *Synechococcus* sp. strain PCC 7002 in each medium was calculated in order to optimize the nitrate concentration to obtain a suitable combination of biomass production and glycogen content, as shown in Figure 3d. The peak of glycogen production was 3.5 g L^−1^ in brackish water with 13 and 15 mM nitrate, 3.0 g L^−1^ in seawater with 15 mM nitrate, or 1.8 g L^−1^ in freshwater with 9 mM nitrate (Figure 3d). Glycogen production in *Synechococcus* sp. strain PCC 7002 maintained the same level in seawater and half of the level in freshwater compared with the level achieved in brackish water.

## Discussion

Cyanobacterial glycogen is remarkable carbon source for bioethanol production by yeast fermentation [5]. As shown in Figure 2a, glycogen accumulated under high light intensity and high CO_2_ concentration. *In vitro* and *in situ* kinetic experiments have revealed that cyanobacterial glycogen synthesis is regulated by adenosine diphosphate (ADP)-glucose pyrophosphorylase (AGPase) activity, which is enhanced by 3-phosphoglycerate (3-PG) accumulation and inhibited by inorganic phosphorus accumulation [27]. Therefore, 3-PG might be accumulated by the increase in light intensity and CO_2_ concentration, which would lead to glycogen accumulation in *Synechococcus* sp. strain PCC 7002.

The glycogen production of *Synechococcus* sp. strain PCC 7002 was examined under different nitrate additions in a brackish water medium (Figure 3a). As shown in Additional file 1: Figure S1, cell growth in brackish water media under 9 and 15 mM nitrate supplies were inhibited by nitrogen limitation. Under nitrogen-limiting conditions, biomass production would be strongly inhibited due to the relatively low photosynthesis efficiency, expecting that light-harvesting proteins (such as phycobiliproteins) would be degraded to compensate for the insufficient nitrogen availability [28]. On the other hand, the glycogen content in cyanobacteria is accumulated by nitrogen depletion [16-20]. Since lower initial nitrate supplies caused faster nitrate depletion (as shown in Additional file 2: Figure S2), glycogen content increased gradually with a decrease in initial supplied nitrate as shown in Figure 3a.

In addition, glycogen production was influenced by salinity in medium as shown in Figure 3a-d. Glycogen production in seawater was a little lower than brackish water, which was caused by the lower glycogen content (Figure 3a,b,d). Glycogen content in seawater would be reduced by the accumulation of osmolytes, such as glucosylglycerol, glucosylglycerate, and sucrose in *Synechococcus* sp. strain PCC 7002, with an increase in sodium chloride concentration [16,17,30]. Also, the decline of glycogen production in freshwater was due to lower biomass production (Figure 3a,c,d). High cell density in *Synechococcus* sp. strain PCC 7002 could not be obtained in the freshwater medium.

The biomass production and α-polyglucan production in various cyanobacteria and microalgae are summarized in Table 1. The highest biomass production (7.2 g L^−1^) and α-polyglucan production (3.5 g L^−1^) from *Synechococcus* sp. strain PCC 7002 under the optimal conditions with the brackish water medium are higher than that reported by other studies [19,21,22,25,30-36]. In addition, glycogen production of *Synechococcus* sp. strain PCC 7002 in a seawater and freshwater environment is greater than or similar with other cyanobacteria and microalgae as shown in Table 1. Therefore, *Synechococcus* sp. strain PCC 7002 would not only provide glycogen from coastal seawaters without the need for freshwater resources, but also can produce the highest level of α-polyglucan among microalgae and cyanobacteria in wide salinity conditions.

**Table 1:** Production of biomass and α-polyglucan by microalgae and cyanobacteria under phototrophic condition

**Species**	**Biomass production (g-dry biomass L**^**−1**^**)**	**α-polyglucan production (g L**^**−1**^**)**	**α-polyglucan content (% of dry biomass)**	**Light intensity (μmol photons m**^**−2**^ **s**^**−1**^**)**	**Nitrogen source**	**Carbon source**	**Medium**	**Reference**
*Porphyridium* sp. UTEX 637	5.6	0.36	6.7	300	10 mM KNO_3_	1.5–2% CO_2_ aeration	Seawater	[30]
*Porphyridium aerugineum*	5.0	0.63	12.7	300	5.2 mM NaNO_3_	1.5–2% CO_2_ aeration	Freshwater	
*Tetraselmis subcordiformis*	5.7	2.7	47.8	200	11 mM KNO_3_	3% CO_2_ aeration	Seawater	[31]
*Chlorella vulgaris* CCAP 211/11B	2.4	1.3	55.0	300	6 mM KNO_3_	2% CO_2_ aeration	Freshwater	[32]
*Arthrospira maxima* SOSA 18	0.95	0.91	70.0	50	No addition	200 mM HCO_3_^−^	High sodium water^a^	[21]
*Arthrospira platensis* NIES-39	1.6	1.0	63.0	700	3 mM NaNO_3_	200 mM HCO_3_^−^	High sodium water^a^	[19]
*Arthrospira platensis* NIES-46	1.1	0.58	53.0	50	No addition	200 mM HCO_3_^−^	High sodium water^a^	[33]
*Anabaena variabilis* ATCC 29413	0.3	0.08	26.7	50	No addition	1.5% CO_2_ aeration	Freshwater	[22]
*Gloeocapsa alpicola* CALU 743	N.D.	0.60	N.D.	220	4 mM KNO_3_	2% CO_2_ aeration	Freshwater	[34]
*Plectonema boryanum* ATCC 18200	0.34	0.08	22.0	100	0.5 mM Ca(NO_3_)_2_•4H_2_O	Air	Freshwater	[35]
*Synechocystis* sp. PCC 6701	N.D.	0.46	N.D.	40	No addition	1% CO_2_ aeration	Freshwater	[36]
*Synechococcus* sp. PCC 7002	N.D.	0.33	N.D.	2500	11 mM NaNO_3_	1% CO_2_	Brackish water	[25]
	7.2	3.5	49.8	600	15 mM NaNO_3_	2% CO_2_	Brackish water	This work
	7.7	3.0	38.7	600	15 mM NaNO_3_	2% CO_2_	Seawater	
	2.8	1.8	62.2	600	9 mM NaNO_3_	2% CO_2_	Freshwater	

N.D.: Not determined.

^a^High sodium water indicates SOT medium [19].

To further improve glycogen productivity in *Synechococcus* sp. strain PCC 7002, the glycogen accumulation rate should be accelerated through metabolic engineering. According to Kumaraswamy *et al*., the intracellular glycogen content in *Synechococcus* sp. strain PCC 7002 is positively correlated with the expression level of the NAD^+^-dependent glyceraldehyde 3-phosphate dehydrogenase (GAPDH-1) gene under photoautotrophic conditions [15]. Accordingly, glycogen productivity in *Synechococcus* sp. strain PCC 7002 may be further improved by a combination of the optimization of growth conditions and the overexpression of GAPDH-1. Glycogen produced by *Synechococcus* sp. strain PCC 7002 in this study was converted to ethanol by yeast fermentation (Additional file 3: Figure S3). The enhancement of glycogen production by *Synechococcus* sp. strain PCC 7002 would contribute to biofuel production.

## Conclusions

*Synechococcus* sp. strain PCC 7002 which combines a wide salinity tolerance and high glycogen production capacity could become an important carbon source for the development of biofuels and bio-based chemicals production. The glycogen productivity of *Synechococcus* sp. strain PCC 7002 would be further enhanced through genetic engineering or metabolic engineering in the next step, which could accelerate the glycogen accumulation rate under nitrogen depletion.

## Methods

### Microorganism and growth conditions

The cyanobacteria *Synechococcus* sp. strain PCC 7002 was obtained from the Pasteur Culture Collection (Paris, France). Cells were pre-cultured in 500 mL Erlenmeyer flasks containing 250 mL of modified medium A (3.0 g L^−1^ NaNO_3_, 50 mg L^−1^ KH_2_PO_4_, 18 g L^−1^ NaCl, 5.0 g L^−1^ MgSO_4_•7H_2_O, 0.37 g L^−1^ CaCl_2_•2H_2_O, 0.60 g L^−1^ KCl, 32 mg L^−1^ Na_2_EDTA•2H_2_O, 8.0 mg L^−1^ FeCl_3_•6H_2_O, 34 mg L^−1^ H_3_BO_3_, 4.3 mg L^−1^ MnCl_2_•4H_2_O, 0.32 mg L^−1^ ZnCl_2_, 30 μg L^−1^ MoO_3_, 3.0 μg L^−1^ CuSO_4_•5H_2_O, 12 μg L^−1^ CoCl_2_•6H_2_O, 4.0 μg L^−1^ cobalamin, and 8.3 mM Tris aminomethane, all of which were purchased from Nacalai Teque, Inc., (Kyoto, Japan)) [37] with 100 rpm agitation under continuous illumination at 50 μmol photons m^−2^ s^−1^ for 7 days in air at 30 ± 2 °C in an NC350-HC plant chamber (Nippon Medical and Chemical Instruments, Osaka, Japan). Experiments were carried out in a closed double-deck flask, containing in the first stage 50 mL of 2 M NaHCO_3_/Na_2_CO_3_ buffer with the appropriate pH to obtain the desired CO_2_ concentration [38,39], and containing in the second stage 70 mL of culture medium. NaHCO_3_/Na_2_CO_3_ buffer was exchanged after 4 days to maintain the desired CO_2_ concentration. Pre-cultured cells were inoculated into fresh medium at a dry-based biomass concentration of 0.01 g dry-cell weight L^−1^ (the optical density at 750 nm (OD750) value was 0.04) and cultivated for 7 days at 33 ± 3 °C with 80 rpm agitation. The effects of light intensity and CO_2_ concentration on glycogen production were examined under 50, 300, or 600 μmol photons m^−2^ s^−1^ at 0.04 (atmospheric level), 1, 2, or 4% (v/v) CO_2_ in air. Light intensity was measured in the middle of the medium using an LI-250A light meter (LI-COR, Lincoln, Nebraska, USA) equipped with an LI-190SA quantum sensor (LI-COR). To study the effect of nitrate supply in different salinity media under 600 μmol photons m^−2^ s^−1^ in 2% CO_2_ in air, pre-cultured cells were transferred into 3-types of media with 9 to 35 mM nitrate. : 1) medium A (brackish water medium; salinity at 2.7%), 2) medium A containing 0.075 g L^−1^ MgSO_4_•7H_2_O, 0.036 g L^−1^ CaCl_2_•2H_2_O, 0.04 g L^−1^ K_2_HPO_4_ without NaCl (freshwater medium; salinity at 0.3%), 3) medium A containing 29.2 g L^−1^ NaCl, 7 g L^−1^ MgSO_4_•7H_2_O, 4 g L^−1^ MgCl_2_•6H_2_O, 1.47 g L^−1^ CaCl_2_•2H_2_O, 0.6 g L^−1^ KCl, 0.05 g L^−1^ KH_2_PO_4_ (seawater medium; salinity at 4.0%). Medium salinity were measured with a refractometer (S/Mill-E; Atago Co. Ltd, Tokyo, Japan).

### Analytical methods

Cell growth was monitored by measuring OD750 in a spectrophotometer (UVmini-1240, Shimadzu, Kyoto, Japan) [29]. Cell concentration was shown as dry-cell weight during cultivation and was converted using a pre-established calibration between dry-cell weight and optical density of cell suspension (1.0 OD750 equals approximately 0.32 g dry-cell weight L^−1^). Dry-cell weight was determined by centrifugation of serial diluted cell-suspension (6,300 × g for 2 minutes at 25 °C), washing the pellet once with 0.3 M ammonium carbonate and lyophilization.

Glycogen content and concentration were determined by high performance liquid chromatography (HPLC) (Shimadzu, Kyoto, Japan) using a size exclusion HPLC column (OHpak SB-806 M HQ; Shodex, Tokyo, Japan) and a reflective index detector (RID-10A; Shimadzu, Kyoto, Japan) [40]. Glycogen was extracted from the dried cells by the modified method of Ernst and Böger [22]. Glycogen productivity (g L^−1^ d^−1^) was estimated by dividing glycogen production by cultivation time. Experimental data were means of triplicate samples and error bars in the figures indicate the standard deviation.

## Abbreviations

3-PG: 3-phosphoglycerate; ADP: Adenosine diphosphate; AGPase: ADP-glucose pyrophosphorylase; HPLC: High liquid chromatography; OD: Optical density; SD: Standard deviations.

## Competing interests

The authors declare that they have no competing interests.

## Authors’ contributions

SA designed the study and wrote the manuscript. AN performed the experiments and analyzed the data. SHH revised manuscript. TH designed the study and revised manuscript. JSC and AK coordinated the study. All authors read and approved the final manuscript.

## Supplementary Material

Additional file 1: Figure S1 Growth curve under different nitrate supplies in brackish water medium. Cells were cultivated under 600 μmol photons m^−2^ s^−1^ and 2% CO_2_ condition with 9 to 27 mM nitrate supplies. Error bars indicate standard deviations (SD) of three replicated experiments. In some data points, error bars obtained by three replications are smaller than symbols. Click here for file

Additional file 2: Figure S2 Nitrate consumption under different nitrate supplies in brackish water medium. Nitrate concentrations were determine according to method proposed by American Public Health Association [41]. Cells were cultivated under 600 μmol photons m^−2^ s^−1^ and 2% CO_2_ from 35 to 9 mM nitrate supplies. Error bars indicate standard deviations (SD) of three replicated experiments. In some data points, error bars obtained by three replications are smaller than symbols. Click here for file

Additional file 3: Figure S3 Ethanol production from glycogen extracts of *Synechococcus* sp. strain PCC 7002 following yeast fermentation. Ethanol was produced from glycogen extracts of *Synechococcus* sp. strain PCC 7002 by *Saccharomyces cerevisiae* MT8-1 in the presence of 0.3 U L^−1^ α-amylase and 0.1 U L^−1^ glucoamylase. Glycogen extracts of *Synechococcus* sp. strain PCC 7002 were prepared as described in Methods and then adjusted to pH 7.0 using 98% H_2_SO_4_ (w/w). *S. cerevisiae* MT8-1 cells were grown aerobically in 1-L Erlenmeyer flasks containing 500 mL YPD medium (10 g L^−1^ yeast extract, 20 g L^−1^ peptone, and 20 g L^−1^ glucose) at 30°C with 150 rpm agitation for 48 hours, and then collected by centrifugation at 5,000 × g for 3 minutes at 25°C, washed twice with distilled water, and then inoculated into 50 mL YPG medium (10 g L^−1^ yeast extract, 20 g L^−1^ peptone, 0.1 M phosphate buffer adjusted to pH 6.0, 10 mM disodium EDTA, and 10 g L^−1^*Synechococcus* sp. strain PCC 7002 glycogen extract). Ethanol production was performed at 30°C and an agitation speed of 500 rpm in 100-mL closed bottles equipped with a bubbling CO_2_ outlet and a stir bar under oxygen-limited conditions. Agitation speed was maintained with a magnetic stirrer (VARIOMAG Telesystem; Thermo Fisher Scientific, Waltham, Massachusetts, United States). Click here for file
