# Supplementary figures and images for: Glycogen production for biofuels by the euryhaline cyanobacteria Synechococcus sp. strain PCC 7002 from an oceanic environment
Source: Biotechnol Biofuels. 2014 Jun 11;7:88. doi: 10.1186/1754-6834-7-88 (PMC4067375; doi:10.1186/1754-6834-7-88)

Figure S1

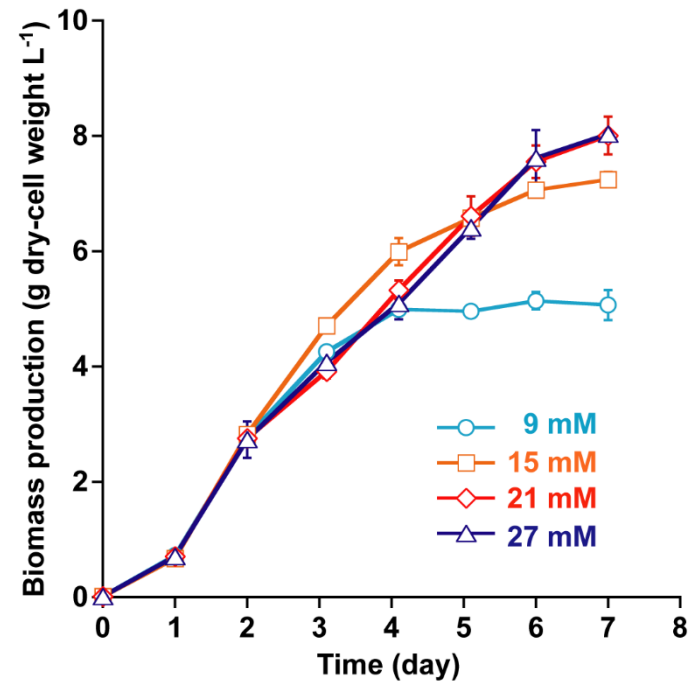

Supplement: Additional file 1: Figure S1 — Growth curve under different nitrate supplies in brackish water medium. Cells were cultivated under 600 μmol photons m−2 s−1 and 2% CO2 condition with 9 to 27 mM nitrate supplies. Error bars indicate standard deviations (SD) of three replicated experiments. In some data points, error bars obtained by three replications are smaller than symbols. [file 1754-6834-7-88-S1.pdf]

Figure S2

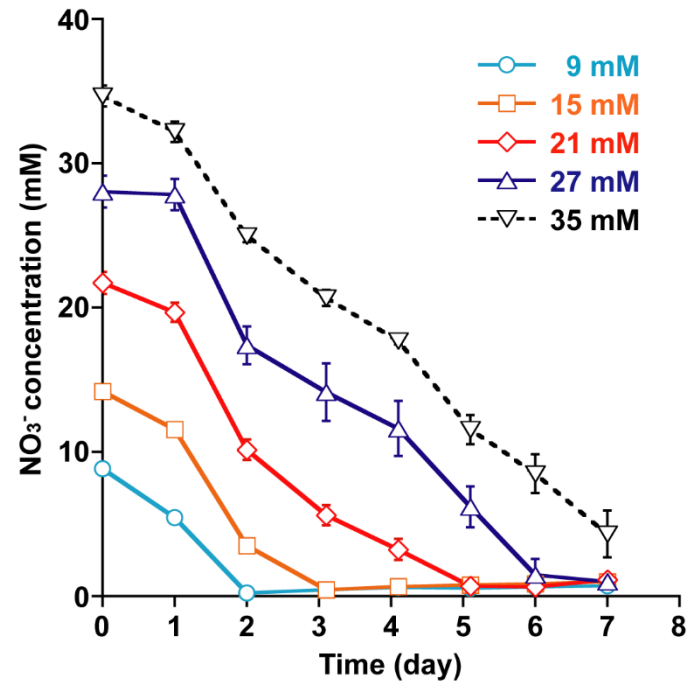

Supplement: Additional file 2: Figure S2 — Nitrate consumption under different nitrate supplies in brackish water medium. Nitrate concentrations were determine according to method proposed by American Public Health Association [41]. Cells were cultivated under 600 μmol photons m−2 s−1 and 2% CO2 from 35 to 9 mM nitrate supplies. Error bars indicate standard deviations (SD) of three replicated experiments. In some data points, error bars obtained by three replications are smaller than symbols. [file 1754-6834-7-88-S2.pdf]

Figure S3

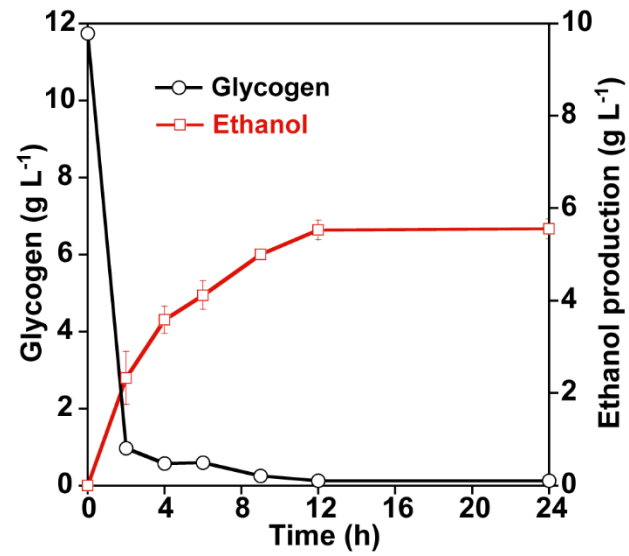

Supplement: Additional file 3: Figure S3 — Ethanol production from glycogen extracts of Synechococcus sp. strain PCC 7002 following yeast fermentation. Ethanol was produced from glycogen extracts of Synechococcus sp. strain PCC 7002 by Saccharomyces cerevisiae MT8-1 in the presence of 0.3 U L−1 α-amylase and 0.1 U L−1 glucoamylase. Glycogen extracts of Synechococcus sp. strain PCC 7002 were prepared as described in Methods and then adjusted to pH 7.0 using 98% H2SO4 (w/w). S. cerevisiae MT8-1 cells were grown aerobically in 1-L Erlenmeyer flasks containing 500 mL YPD medium (10 g L−1 yeast extract, 20 g L−1 peptone, and 20 g L−1 glucose) at 30°C with 150 rpm agitation for 48 hours, and then collected by centrifugation at 5,000 × g for 3 minutes at 25°C, washed twice with distilled water, and then inoculated into 50 mL YPG medium (10 g L−1 yeast extract, 20 g L−1 peptone, 0.1 M phosphate buffer adjusted to pH 6.0, 10 mM disodium EDTA, and 10 g L−1Synechococcus sp. strain PCC 7002 glycogen extract). Ethanol production was performed at 30°C and an agitation speed of 500 rpm in 100-mL closed bottles equipped with a bubbling CO2 outlet and a stir bar under oxygen-limited conditions. Agitation speed was maintained with a magnetic stirrer (VARIOMAG Telesystem; Thermo Fisher Scientific, Waltham, Massachusetts, United States). [file 1754-6834-7-88-S3.pdf]
